# Head and neck cancer cells and xenografts are very sensitive to palytoxin: decrease of c-jun n-terminale kinase-3 expression enhances palytoxin toxicity

**DOI:** 10.1186/1476-4598-12-12

**Published:** 2013-02-14

**Authors:** Tibor Görögh, László Bèress, Elgar S Quabius, Petra Ambrosch, Markus Hoffmann

**Affiliations:** 1 Department of Otorhinolaryngology- Head and Neck Surgery, Section of Experimental Oncology, University of Kiel Schleswig-Holstein, Kiel, 24105, Germany; 2 Immunology and Rheumatology, Section of Peptide Chemistry Hannover Medical School (MHH), Hannover, 30625, Germany; 3 Department of Immunology, University of Kiel Schleswig-Holstein, Kiel, 24105, Germany

**Keywords:** Palytoxin, Anti-tumoral effect, Head and neck carcinoma, Xenografts, JNK3

## Abstract

**Objectives:**

Palytoxin (PTX), a marine toxin isolated from the Cnidaria (zooanthid) *Palythoa caribaeorum* is one of the most potent non-protein substances known. It is a very complex molecule that presents both lipophilic and hydrophilic areas. The effect of PTX was investigated in a series of experiments conducted in head and neck squamous cell carcinoma (HNSCC) cell lines and xenografts.

**Materials and methods:**

Cell viability, and gene expression of the sodium/potassium-transporting ATPase subumit alpha1 (ATP1AL1) and GAPDH were analyzed in HNSCC cells and normal epithelial cells after treatment with PTX using cytotoxicity-, clonogenic-, and enzyme inhibitor assays as well as RT-PCR and Northern Blotting. For xenograft experiments severe combined immunodeficient (SCID) mice were used to analyze tumor regression. The data were statistically analyzed using One-Way Annova (SPSS vs20).

**Results:**

Significant toxic effects were observed in tumor cells treated with PTX (LD50 of 1.5 to 3.5 ng/ml) in contrast to normal cells. In tumor cells PTX affected both the release of LDH and the expression of the sodium/potassium-transporting ATPase subunit alpha1 gene suggesting loss of cellular integrity, primarily of the plasma membrane. Furthermore, strong repression of the c-Jun N-terminal kinase 3 (JNK3) mRNA expression was found in carcinoma cells which correlated with enhanced toxicity of PTX suggesting an essential role of the mitogen activated protein kinase (MAPK)/JNK signalling cascades pathway in the mechanisms of HNSCC cell resistance to PTX. In mice inoculated with carcinoma cells, injections of PTX into the xenografted tumors resulted within 24 days in extensive tumor destruction in 75% of the treated animals (LD50 of 68 ng/kg to 83 ng/kg) while no tumor regression occurred in control animals.

**Conclusions:**

These results clearly provide evidence that PTX possesses preferential toxicity for head and neck carcinoma cells and therefore it is worth further studying its impact which may extend our knowledge of the biology of head and neck cancer.

## Background

Palytoxin (PTX), a toxin isolated from the marine Cnidaria *Palythoa caribaeorum,* has a molecular weight of 3300 dalton and was first isolated by Moore and Scheuer [1]. Recently, it was demonstrated that also Dinoflagellates of the *Ostreopsis* genera produce this compound and analogues [2]. PTX is one of the most toxic non-peptidic natural products known to date. From a chemical perspective, it is a large, very complex molecule with a long polyhydroxylated and partially unsaturated aliphatic backbone, containing 64 chiral centers [3]. In contrast to most cytotoxins, PTX exerts its activity extracellularly by altering ion equilibria in biological systems [4]. PTX displays an extraordinary level of cytotoxic activity on a variety of cell lines and it develops a wide spectrum of pharmacological effects such as cellular disruption, binding of the toxin to its receptor [5], and modulation of protein kinase signalling cascades [6]. Other studies highlight the cytoskeleton as an early target for the toxic effects of PTX and its analog ostreocin-D on intestinal [7] and neuroblastoma cells [8].

Most studies focused on the function and mechanism of PTX which acts through the Na^+^, K^+^-ATPase [9], H^+^, K^+^ –ATPase [10], interaction with ion channels, and binding reaction to the Na/K pump [5,11,12]. PTX targets the Na^+^, K^+^ ATPase via binding and locking it in a position allowing passive transport of both the sodium and potassium ions, thereby destroying the ion gradient that is essential for most cells [13]. The Na^+/^K^+^-transporting ATPase subunit alpha-1 is an enzyme that in humans is encoded by the ATP1AL1 gene [14]. Dysfunctions in the Na^+^, K^+^-ATPase pump may also affect other secondary ion transporters, including Na^+^, Ca^2+^ exchange, leading to membrane depolarization [15]. The PTX-induced membrane depolarization interferes with some vital functions of the cells. Altered concentration of intracellular cations, in particular calcium increase, is generally associated with cell death [16]. As a consequence of alterations in ion gradients, many modifications of cytosolic proteins occur. Thus, PTX causes modulation of mitogen-activated protein kinase (MAPK) cascades [6] and stimulates JNK activation in mouse 3T3 fibroblasts [17]. It was suggested that PTX is also capable of perturbing growth regulatory systems by down-regulation of epidermal growth factor (EGF) binding through a protein kinase C-independent pathway. Inhibition of EGF binding is highly dependent on extracellular Na [18,19]. On the other hand, PTX was found to be a non-12-O-Tetradecanoylphorbol 13-acetate (TPA)-type tumor promoter [20,21] inducing a signal pathway leading to activation of stress-activated protein kinases (SAPK) JNK important for signal transduction pathways [22].

The effects shown in various animal species after PTX treatment *in vivo*[23] have been confirmed also by *in vitro* studies. Initial experiments were performed on excitable cells of various origin, from muscles and nervous system, and in those cases PTX could be distinguished from other toxins on the basis of severe effects including contractile action on vascular smooth muscle [24], increase in cation permeability and depolarization [25] as well as plasma membrane lysis [16].

The objective of this study was to analyze the effects of PTX on several HNSCC cell lines in comparison to healthy epithelial cells and determine how sensitive xenografted tumors are to this toxin. We also focused on signalling complexes and molecular compounds such as the MAPK/JNK signalling cascades pathway aiming to understand the underlying molecular mechanisms responsible for the difference in PTX toxicity between normal- and HNSCC cells.

## Methods

### Human cell lines

For the *in vitro* experiments cell lines derived from human HNSCC of different localizations were used: oropharynx, (UKHN-1), esophagus (UKHN-2), tongue (UKHN-3), and tonsil (UKHN-6). All cell lines were authenticated by single tandem repeat DNA typing (DSMZ, Braunschweig, Gemany). Five human epithelial cell cultures, derived from normal mucosa of the pharynx and the larynx served as controls. Prior to analyses all cell lines were negatively tested for mycoplasma. The normal mucosa biopsies were retrieved during surgery after written patient consent was obtained, in accordance with the Ethical Commission of the University of Schleswig-Holstein, Campus Kiel, subjected to the Helsinki Declaration, revised 1983 (No. AZ D 438/10). The carcinoma cells were grown in minimum essential medium with 10% (v/v) fetal calf serum (Biochrom, Berlin, Germany) at 37°C in 5% CO_2_ humidified atmosphere. Normal epithelial cells were grown in SFM-medium (Life Technologies, Inc. Eggenstein, Germany) under same conditions as the carcinoma cells.

### RT-PCR and Northern blot analysis

Total RNA of tumor and normal cells was extracted using the RNeasy Kit (Qiagen, Hilden, Germany) and 100ng total RNA were used for cDNA synthesis using MMLV reverse transcriptase following the manufacturer’s instruction (Life Technologies, Inc.). For RT-PCR analysis, the cDNA was amplified for 30 cycles using the sense 5’-AGATTCCGAGAAGAAGACCA-3’ and anti-sense 5’-GCTGGGGCTCAGACTCCCCCGTGAGA-3’ oligonucleotides specific for the ATP1AL1 gene (annealing temperature 55°C) and the sense 5’-CCAGCCGAGCCACATCGC-3’ and anti-sense 5’-ATGAGCCCCAGCCTTCTCCAT-3’ oligonucleotides specific for the GAPDH gene (annealing temperature 55°C). For Northern hybridization a 535 bp probe was designed using the sense primer 5‘-CTGTATTGCAGCTAAGCTC-3‘ and the anti-sense primer 5‘-CTACATCTTCAAGGGTCTG-3’ (positions 1799–1817 and 2315–2333) of the JNK3-mRNA (GenBank accession no. NM_002753) which was labelled by chemoluminescence (Roche Diagnostics, Germany). Twenty microgram total RNA from tumor cells and normal epithelial cells were separated on 1% agarose gel. After transfer of the RNA onto nylon membrane both hybridization- and detection procedures were carried out according to the manufacturer’s instructions.

### Isolation of PTX

PTX was isolated chromatographically from the marine Cnidaria (zooanthid) *Palythoa caribaeorum* and purified as we described earlier [26]. Purified PTX was lyophilized and stored at −20°C.

### Cytotoxicity assay

Quantification of cell death and cell lysis was based on the measurement of LDH activity released from the cytosol of damaged cells into the supernatant using a non-radioactive LDH detection kit (Roche Diagnostics, Germany). Cells grown to a monolayer were incubated for 24 h in the presence (1-4 ng/ml) or absence of PTX. After centrifugation at 250xg for 10 min. the cell-free culture supernatants were collected from PTX treated -and untreated cells and incubated according to the manufacturers instruction. To calculate percent cytotoxicity appropriate controls were measured in each experiment. Absorbance was measured at 492 nm and 620 nm using an ELISA reader (Dynatech MR5000, Denkendorf, Germany).

### Clonogenic assay

At day 0, HNSCC cells and normal epithelial cells were plated in duplicate in 6-well plates. One week later, after cells had reached confluency, they were incubated for 24h at various PTX-concentrations (0.5 ng/ml to 10 ng/ml). Subsequently, cells were washed with PBS, fixed in ethanol and stained with crystal violet (0.1% crystal violet in 20% ethanol). Stained cells were measured by microscopic counting randomly choosing at least ten middle power magnification (x200) fields. Mean values and standard deviation (SD) were calculated.

### JNK3 inhibitory assay

Pyrazolourea, a selective inhibitor of JNK3 (IC50 = 7 nM [27]) was obtained from Merck/Calbiochem, Germany. Normal epithelial cells were seeded in 6-well plates and cultured until confluent. The cells were incubated with pyrazolourea at concentrations ranging from 20 nM to 100 nM for 3 hours to inhibit the JNK3 protein kinase. Subsequently, cells were exposed to 6 ng/ml PTX for 24 hours. Finally, cell survival was determined using the crystal violet assay.

### Animal experiments

SCID bg/bg mice were obtained from Charles River (Sulzfeld, Geramny) aged 10 to 12 weeks. For the carcinogenicity experiments a group of tumor-free mice (n=8) was treated by subcutaneous (*sc*) injection of 0.5ng PTX in a volume of 20 μl PBS/day for 5 days. Subsequently,the animals were observed over a period of 8 months. After that period the internal organs such as liver, kidneys, and spleen were examined histopathologically.

For the therapeutic response study mice were divided into three tumor groups and a control group. In the tumor groups (*n*=24) mice were injected *sc* in flank with one million tumor cells (a mix of UKHN-1, UKHN-2, and UKHN-3 cell lines 1:1:1). Two weeks after tumor cell inoculation, one tumor group (*n*=8) received intratumoral injections (1,5 ng PTX dissolved in 20 μl PBS ) every three days over a period of further 24 days, another tumor group (n=8) received intraperitoneal (*ip*) injections of the same dose every three days and the third tumor group (n=8) received intratumoral injections of PBS (20 μl). Tumor size was measured three times per week with a calliper. After the therapeutic response study residual tumors as well as liver, kidneys, and spleen were examined histopathologically.

In the control group (*n*=8) mice were each injected *sc* in their flanks with one million normal epithelial cells. This group was also observed over the period of further 24 days in order to assure that no tumor growth occurred. The experiments in SCID mice were approved by the Ministry of Environment, Nature and Agriculture of Schleswig-Holstein, Germany. However, with a restriction in animal numbers to be used that led us to use the above mentioned combination of HNSCC cells, rather than using each cell line individually.

### Immunohistochemistry

For immunohistochemical evaluation 8 subcutanous xenograft tumors were used. One of them was analysed prior to the start of the intratumoral PTX treatment, one after 8 and 16 days respectively, and the remaining five tumors 24 days after PTX treatment. The tumors were fixed in formalin and embedded in paraffin. Deparaffinized sections (5 μm) were stained with hematoxilin and eosin.

### Statistical analysis

Statistical analysis of the data was performed by means of One-Way ANOVA (SPSS vs 20). Data were considered statistically significant if p ≤ 0.05.

## Results

### HNSCC cells are more sensitive to PTX than normal cells

**Figure 1:**
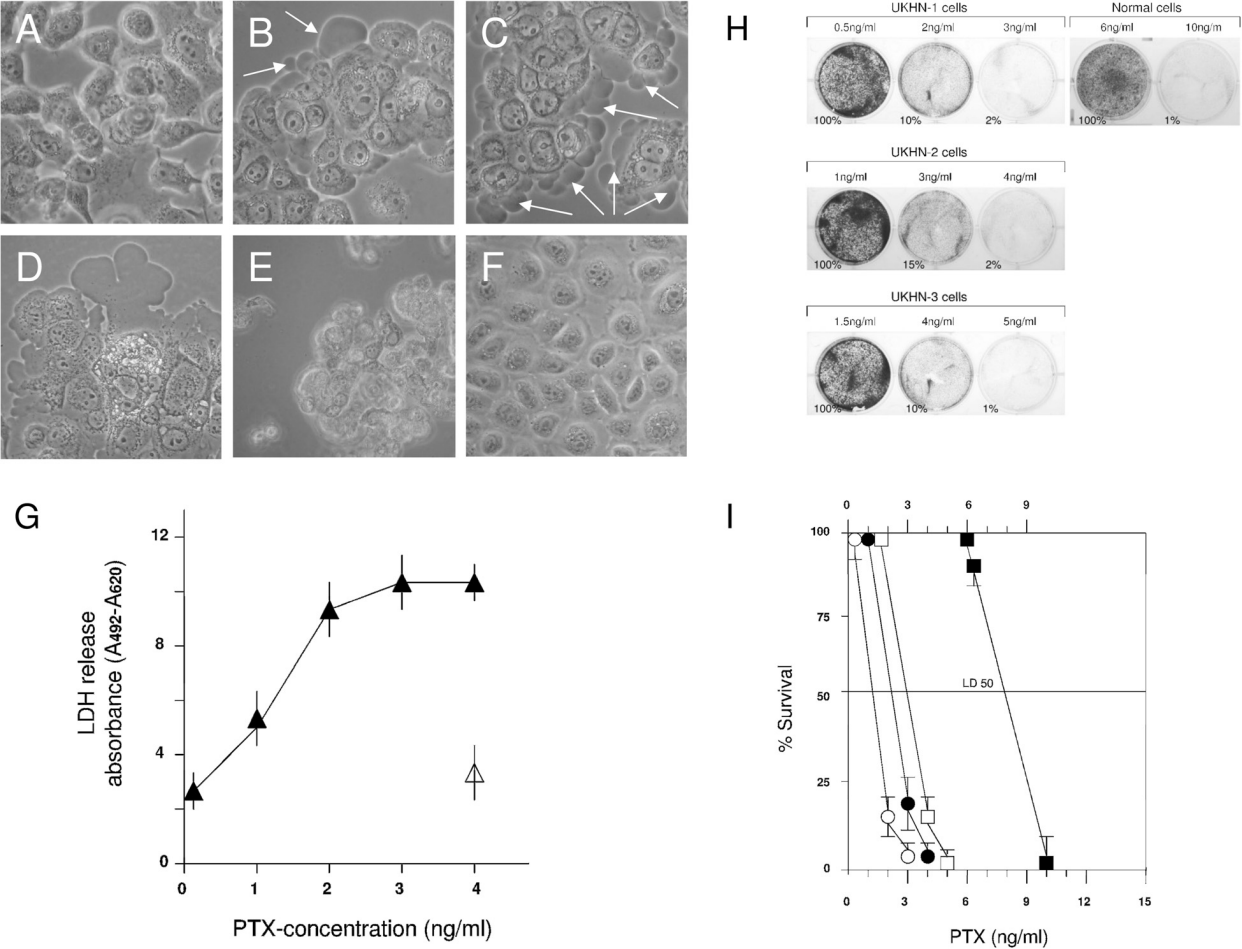
**Analysis of cell morphology and effect of PTX on high**-**density cell cultures. **(**A**) Typical morphological appearance of PTX untreated UKHN-6 cells. (**B-E**) Dose-dependent morphological alterations of the carcinoma cells. (**B**) At 1 ng/ml PTX the cells begin to disintegrate, reflected by initiation of cellular swelling (arrows). (**C**) Swelling process is advanced in all cells at 2 ng/ml PTX. (**D**) Cellular damage is spreading throughout the cells at 3 ng/ml PTX, shown by high grade of flattening. (**E**) At 4 ng/ml PTX cells are detached from the culture surface and are completely destroyed (**F**), whereas no morphological changes are recognizable in normal epithelial cells at this concentration (**F**). (**G**) Release of LDH from carcinoma cells (▲) and normal epithelial cells (Δ) shown in Fig. B-E and F respectively. (**H** and **I**) Effect of PTX on high-density cell cultures utelising cytotoxicity and clonogenic assays. UKHN-1 (○), UKHN-2 (●), and UKHN-3 (□) tumor cells, and normal epithelial cells (■) were used. (**H**) Cell survival was determined by using the crystal violet assay. Percentages indicate the amount of surviving cells after treatment with different PTX concentrations. (**I**) Percent survival of PTX treated cells shown in H. Data represent the mean ± SD of triplicate experiments.

Prior to the clonogenic and cytotoxicity assays the effect of PTX on the morphology and proliferation rate of the HNSCC cell lines (UKHN-1,2,3 and UKHN-6) was determined in comparison to normal epithelial cells. All carcinoma cells exhibited similar morphological changes which are exemplarily shown for UKHN-6 cells (Figure 1A-E). In the absence of PTX, the culture consisted of small, polygonal cells (Figure 1A). Beginning with the application of PTX, typical signs of cellular damage, such as pleomorphism, prominent nuclei, and cytosolic alterations were observed. Morphologic characteristics of carcinoma cells in the presence of different PTX concentrations changed in a dose-dependent manner. The first evidence of cell damage was cellular swelling at 1 ng/ml PTX (Figure 1B) which was increased with increasing PTX concentration (2 ng/ml; Figure 1C). At 3 ng/ml PTX carcinoma cells had structurally changed in size, shape, and appearance while typical features such as pleomorphic nuclei and prominent nucleoli still remained (Figure 1D). Exposure to 4 ng/ml led to complete destruction of carcinoma cells (Figure 1E). In contrast, no morphological changes were observed in normal epithelial cells at this PTX concentration (Figure 1F). Notably, these morphological responses correlated with the energy metabolisms of the cells as shown by LDH release assay (Figure 1G). 

To further elucidate the effect of PTX we analyzed additional HNSCC cell lines originated from tumors of different anatomical locations, including oropharynx (UKHN-1), esophagus (UKHN-2), and tongue (UKHN-3) (Figure 1H and 1I). The median lethal dose, LD50, was reached at concentrations of 1.2 ng/ml (UKHN-1) and 3.0 ng/ml (UKHN-3) respectively. Based on these LD50 values, carcinoma cells would be expected to be 2.5 to 6.0 times more sensitive to PTX than normal cells. Among the carcinoma cells tested, the UKHN-1 oropharyngeal squamous cell carcinoma cells showed the highest sensitivity to PTX, suggesting some differences of HNSCC cells in sensitivity to PTX. Collectively, the cytotoxic experiments indicate that PTX possesses preferential toxicity for HNSCC cells without causing any damage to healthy epithelial cells under similar treatment condition

### Effect of PTX on solid tumor xenografts

A group of tumor-free mice (n=8) were treated by *sc* injectio*n* with PTX (0.5 ng/day for 5 days) before beginning the experiments examining the anti-tumor-effect of PTX in tumor-bearing mice. This initial experiment should demonstrate that PTX has no mutagenic effect and does not act as a tumor initiator in mice. After an incubation period of eight months, the injection sites of the animals along with the internal organs such as liver, kidneys, and spleen, were examined, and no evidence of tumor development could be found (one out of 8 animals died spontaneously for unknown reasons) (Table 1). 

**Table 1:** Analysis of the tumorigenic effect of PTX after *sc *administration in SCID mice

**SCID mice**	Single dose of PTX (ng)*	**Frequency of PTX injection**	**Observation time (months)**	**Tumor development**
1	0.5	5	8	no
2	0.5	5	8	no
3	0.5	5	8	no
4	0.5	5	8	no
5	0.5	5	8	no
6	0.5	5	8	no
7	0.5	5	8	no
8	0.5	5	4**	no

*A dose of 0.5ng has been estimated as a concentration at which cell viability still remains.

** One mouse died during the time of observation for unknown reasons.

**Figure 2:**
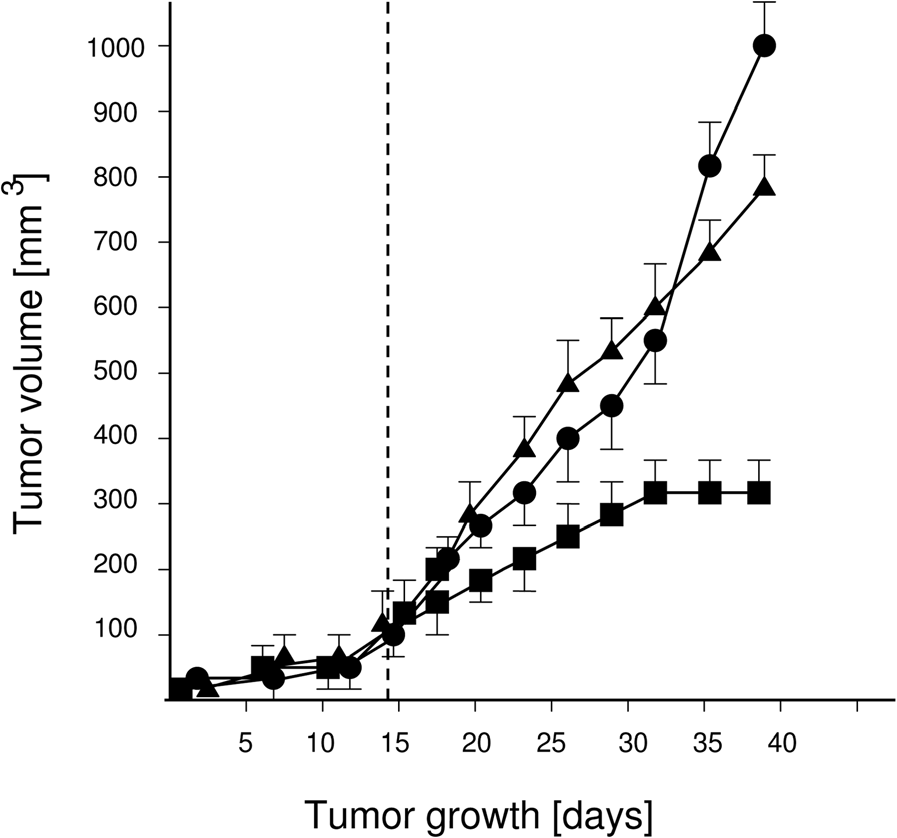
**Tumor development in SCID mice. **Starting on day 14 (vertical broken line), mice received intraperitoneal (●) and intratumoral (■) injections of PTX as well as intratumoral (▲) injections of PBS at three-day-intervals. From day 20 on the differences between the intratumoral and the intraperitoneal treated PTX receiving groups were significant (*P <* 0.05). Similarly, differences were observed when comparing the intratumoral PTX and the intratumoral PBS groups, where tumor sizes were significantly different (p<0.05) from day 20 on. In addition, at all time points measured, there were no significant differences in tumor size between the intratumoral PBS and the ip PTX group (p>0.05). Data represent mean tumor volume ± SD.

In a second experiment the therapeutic efficacy of PTX on solid tumor xenografts was analysed. The carcinoma cells grew subcutaneously as solid tumor xenografts in the mice. The tumors grew quickly, reaching a size of 120 mm^3^ within two weeks. Differences in the course of tumor development between the group receiving intratumoral PTX injections and the groups receiving either *ip* PTX injections or PBS injections are evident (Figure 2). Beginning on day 20 intratumoral administration of PTX was significantly more efficient in tumor reduction when compared to ip PTX injections (p<0.05, for days 20 and 23 and p<0.001 for all other days). Similar results were obtained when comparing intratumoral PTX versus PBS injection, with the PBS injections resulting at no time in different tumor sizes than the tumors in the *ip* PTX treated mice (p>0.05). As shown in Table 2 PTX, administered in doses as low as 68 ng/kg – 83ng/kg extensively inhibited the growth of 6 out of 8 tumors (=75%). In the two remaining tumors only moderate regression was detected. In mice carrying xenotransplants, tumor destruction after intratumoral PTX injection occurred rapidly and progressively without us recognizing signs of distress or abnormal behaviour or any apparent disease symptoms. During the experiment and two weeks after therapy we observed all mice to assure that they did not show any undesired pattern of behavior such as head weaving, suppression of locomotion, reduced climbing activity or decrease in weight in comparison to untreated control animals. Subsequently, the residual tumors were resected and prepared for histological examination. Histological examination of liver, kidneys, and spleen were also carried out in the animals from the therapeutic response study without finding pathological changes in these tissues. 

**Table 2:** Effect of PTX administered into tumors growing in SCID mice

**SCID mice**	Dose of PTX (ng/kg)*	**Number of doses**	Percentage of tumor remission**
1	83	9	100
2	83	9	100
3	68	9	30
4	75	9	100
5	75	9	100
6	75	9	100
7	75	9	30
8	68	9	100

* Each tumor has been treated with 1.5 ng PTX dissolved in 20 μl PBS. Due to different body weight of the mice (18–22 grams) the doses varied from 68 ng/kg to 83 ng/kg.

** One hundred percent tumor remission was defined as the stage at which the entire tumor inside had been destroyed and only the rim of the tumor remained.

### Histological findings on tumors after PTX-treatment

**Figure 3:**
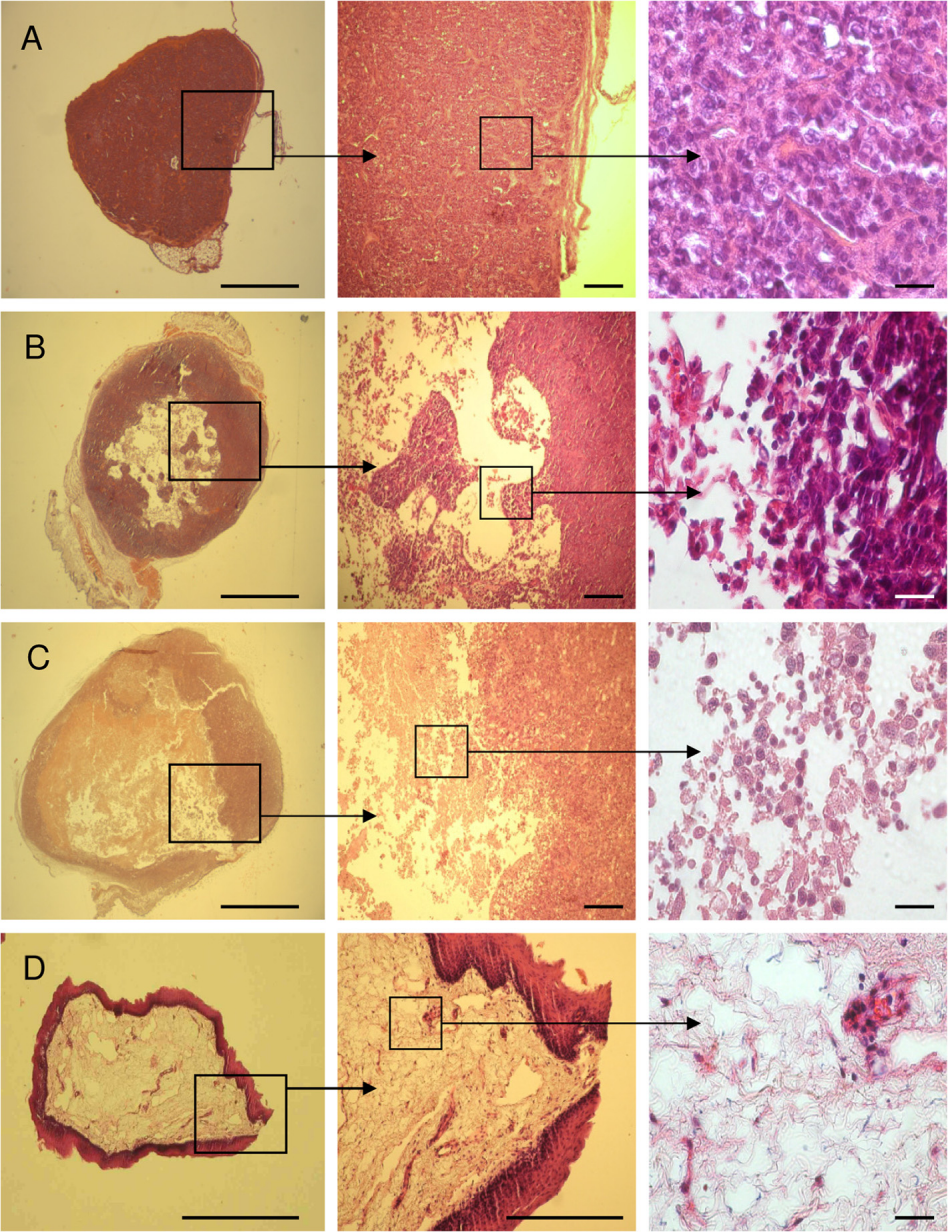
**PTX administration into tumors growing in SCID mice. **(**A**) PTX untreated tumor presenting consistent and extremely dense tumor mass. (**B**) 9 days after beginning of the experiment a loss of tumor mass was already visible. The periphery of tumor necrosis appears to be sharply delineated with loose tumor cell- and lymphoid cell aggregates. (**C**) After 15 days tumor destruction has further progressed, associated with diffuse lymphoid infiltrate. (**D**) The entire tumor inside has been destructed and only the rim of the tumor remained after 24 days. The inside of the tumor is presented by dense collagen fibers with singly scattered lymphoid aggregates. Representative examples of tumor sections from control and PTX-treated group are shown. Scale bars, 2 mm (left), 1 mm (middle), 100 μm (right).

Representative observations regarding the histological appearances of the tumors are presented in Figure 3A-D. The untreated tumor from xenografts showed the typical pattern of squamous cell carcinoma. The tumor cells appeared as densely packed aggregates where the cells surrounded a small lumen separated from the cell surface by a distinct internal limiting membrane (Figure 3A). The resected tumors showed PTX-induced alterations with high grade of necrosis, aggregates of inflammatory cells, peripheral scar formation and granulation tissue at cannula entry sites. The administration of PTX into the tumor at doses of 68 ng/kg-83 ng/kg every three days over a period of 24 days resulted in a reduction of tumor bulk already after 8 days and this phenomenon progressed over the experimental period (Figure 3B-D). Tumor regression occurred by gradual destruction of the tumor inside with obliteration of the tumor tissue architecture. Due to necrotic areas filled with fluid in association with diffuse lymphoid aggregates and remaining collagen fibers, the tumor acquired a considerably softer consistency. At the end of the therapy, only the rim (a fibrous connective tissue capsule which apparently encapsulated and separated the internal tumor mass from the body) remained, the bulk of the tumor was extensively destructed and the tumor appeared as a “deflated balloon” (Figure 3D). At this point the PTX treatment was stopped. During a further period of two weeks with no treatment at all, we found no tumor progression and evaluated the outcome of the intratumoral PTX treatment as positive. 

### PTX-induced molecular alterations

**Figure 4:**
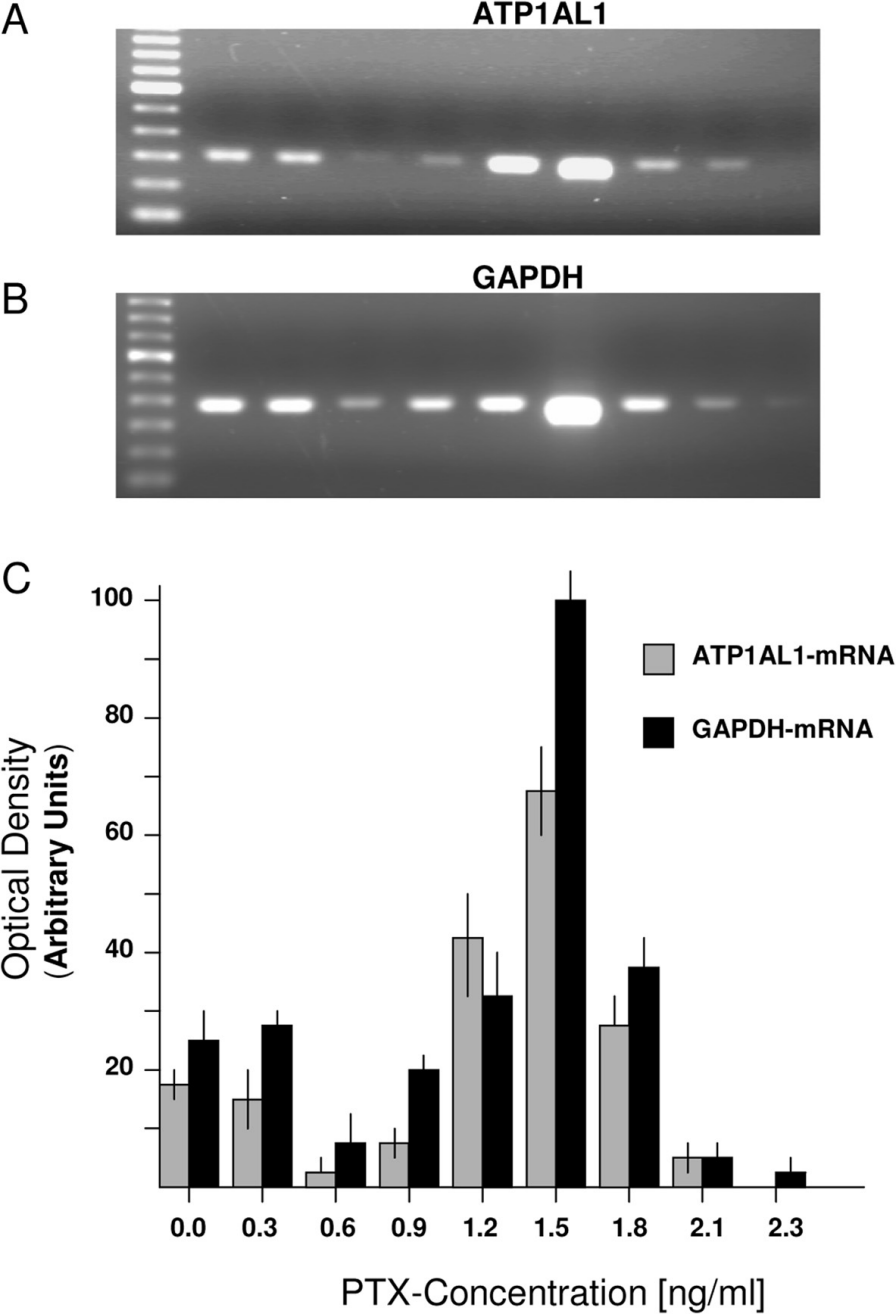
**ATP1AL1- and GAPDH*-*gene expressions in UKHN-2 cells. **(**A** and **B**) Expression profiles of the ATP1AL1- and GAPDH*-*genes during exposure of the tumor cells to PTX of different concentrations. (**C**) Densitometric measurements of the relative gene expression detected by RT-PCR. Data represent mean ± SDs of triplicate measurements.

PTX was applied in vitro to tumor cells, to study the effect of PTX on Na^+^, K^+^ ATPase by measuring ATP1AL1 gene expression. In three independent tumor cell cultures we observed that PTX (0.3 ng/ml) had no effect on ATP1AL1 gene expression. However 0.6 ng/ml PTX led to down-regulation of the gene (Figure 4). Interestingly, down-regulation of ATP1AL1 gene expression did not progress when higher PTX concentrations were used. Quite the contrary occurred: ATP1AL1 gene expression increased, reaching a maximum at 1.5 ng/ml PTX. Additional increases of PTX concentrations in turn caused abrupt decrease in ATP1AL1 gene expression. Similar effects of PTX were seen when analysing GAPDH gene expression (Figure 4). 

### Effect of JNK3 activity on PTX toxicity

**Figure 5:**
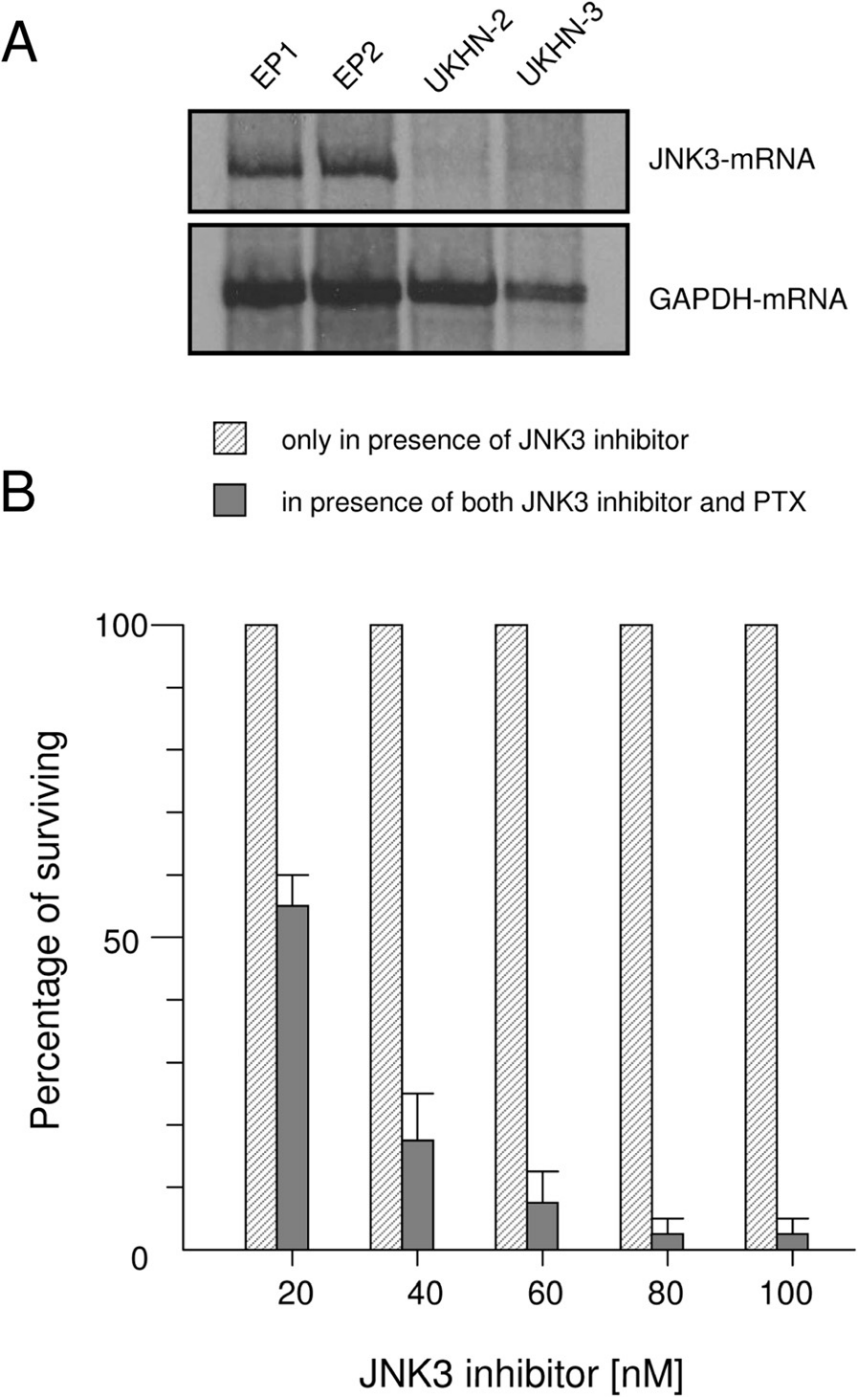
**Repression of JNK3 protein kinase enhances PTX toxicity. **(**A**) Northern Blot hybridization analysis of mRNA in normal epithelial cells (EP1 and EP2) and HNSCC cells (UKHN-2 and UKHN-3) using a JNK3 gene specific cDNA probe. GAPDH specific probe was used as a control for equal loading. (**B**) Human normal nasal epithelial cells were treated with JNK3 protein kinase selective inhibitor at concentrations ranging from 20 nM to 100 nM for 3 hours. Cells of the same origin were then treated under exactly the same conditions, and were subsequently exposed to 6 ng/ml of PTX for 24 hours. Cell survival was determined using the crystal violet assay as demonstrated in Figure 1/H. Percent survival of JNK3 inhibitor-treated cells and those treated with both JNK3 inhibitor and PTX are shown. Data are representative of four independent experiments represent mean ±SD.

By analyzing the MAPK pathway specifically the expression pattern of JNK-mRNA we found strong repression of the JNK3-mRNA expression in tumor cells *vs.* normal cells (Figure 5A). The JNK3 gene encoding protein is a MAPK family member (MAPK10) and is subject to signal transduction pathways in carcinogenesis. To find out whether the JNK3 signaling pathway is directly involved in the mechanism of action of PTX, normal epithelial cells were treated with different concentrations of the cell-permeable pyrazolourea compound that acts as a potent and JNK3-specific inhibitor. Subsequently the cells were exposed to PTX. Finally, cell viability was assessed in comparison to normal epithelial cells treated with PTX but in the absence of the inhibitor. As shown in Figure 5B significant loss of cell viability was already observed at a dose of 20 nM pyrazolourea and PTX mediated toxicity was continuously increased with increasing concentration of pyrazolourea in contrast to non- pyrazolourea -treated cells (p=0.02). Cells remained unaffected when treated with pyrazolourea alone indicating that loss of cell viability is solely attributed to PTX. 

The data presented here provide strong evidence that the repression of JNK3 gene expression is essential for increasing PTX toxicity, suggesting that the MAPK/JNK signalling cascades pathway has a key role in the resistance of HNSCC cells to PTX.

## Discussion

The data presented here show that normal epithelial cells can tolerate higher concentration of PTX without apparent harm than HNSCC cells. The effect of PTX shown on tumor cells suggests that their morphology can be used as an index of PTX toxicity. Morphological change in tumor cells also correlated with LDH release indicating a loss of cellular function, primarily the membrane integrity as would be expected in response to PTX which is known to affect the plasma membrane [15]. It is obvious that many of the pharmacological effects of PTX are attributable to the effect of this substance on trans-membrane ion transfer. PTX has a unique action on the Na^+^,K^+^-ATPase, converting the pump into an ion channel and resulting in K(+) efflux, Na(+) influx and membrane depolarization [25]. PTX can in vitro cause lysis of mouse spleen cells which has been attributed to a PTX-induced increase in cellular calcium levels [28].

The toxicity of PTX in mammals is strongly dependent upon the route of administration [29]. PTX is most toxic by intra venous (*iv*) injection, the LD50 in mice amounted to 0.15 -0.53 μg/kg [1,30]. The PTX toxicity by *ip* administration is lower than that by iv injection, with values of 0.31-1.5 μg/kg being reported for mice [31,32]. PTX is much less toxic orally than after *iv* or *ip* administration. Results from the few existing studies reports an oral LD50 from 510 μg/kg to 767 μg/kg in mice [33,34].

PTX has been described as a tumor promoter [20,21]. This might misleadingly suggest that it is capable of causing tumors. Therefore it is important to note that the basis to classify an agent as a tumor promoter is conditional and is performed only within the context of a two-stage (or multistage) model protocol [35]. The tumor-promoting activity of PTX has been investigated earlier using mouse skin [20]. Thereby, in the first stage carcinogenesis was initiated with the mutagenic compound 7,12-dimethylbenz[a]anthracene (DMBA). In the second stage, repeated application of PTX was performed over a period of several weeks. In mice treated with DMBA and PTX tumor development occurred, but no tumors were observed in animals treated with PTX alone suggesting that PTX treatment alone is not sufficient to generate tumors. To verify this, we performed long-time experiments in which a group of mice were treated daily with 0.5ng PTX for 5 days. By using this low PTX concentration we based our approach on results which showed that PTX concentrations higher than 0.5ng are already toxic to mice [36]. The animals were observed over a period of 8 months without finding evidence of tumor development. Also other studies showed that PTX does not act as a tumor initiator in a Balb/c 3T3 cell transformation assay [37] and it was negative in the Ames mutagenecity test using different strains (TA98, TA100, TA102 and TA1537) [29]. Based on these findings we used PTX to treat tumor xenografts established in SCID mice. Treating these mice with doses as little as 68-83ng/ kg bodyweight we observed rapid and progressive tumor destruction without recognizing any apparent disease symptoms. However, this was only the case when PTX was admistisred intratumoral. None of the mice did show any undesired pattern of behavior during therapy nor during a follow up period of 2 weeks, suggesting that low doses of intratumoral injected PTX might even be beneficial, due to them selectively killing tumor cells rather than normal epithelial cells, but no effects were seen after ip PTX injections.

Alterations in ion gradients induced by PTX at the plasma membrane level play a crucial role in cytotoxic and cell death events [38]. Experimental studies indicated that PTX targets the Na^+^, K^+^ ATPase, and thereby destroys the ion gradient [5,9,12,]. This may lead to a lack of Na^+^, K^+^ ATPase causing dramatic effects on cell function. It is reasonable to hypothesize that a response of the cells to this external influence is the post-production of Na^+^, K^+^ ATPase in order to replace the quantity indispensible for stable cellular conditions. To demonstrate this we analyzed the transcriptional activity of several genes and found that treatment of cells with PTX in fact influences the expression of the ATP1AL1 gene that encodes the Na^+^, K^+^ ATPase. The initial down-regulation and the subsequent progressive up-regulation of this gene is a typical phenomenon of self-regulating, self-protection processes i.e. the ability of the cells to maintain their internal equilibrium due to PTX as an external influencing factor. PTX on the other hand seems to influence the energy metabolism of the cells since we have shown that GAPDH gene expression was also down –and up-regulated as a function of PTX concentration. The expression profiles for both ATP1AL1 and GAPDH genes suggest that PTX induces in the cell lines studied both transcriptional gene suppression and activation. The mechanism involved in such bidirectional transcription process is poorly defined. Recent observations suggest that bidirectional transcription in human cells is an endogenous gene regulatory mechanism whereby small non-coding RNA mediated transcriptional regulation can act in both suppressive [39] and activating manner [40].

PTX stimulates JNK activation through a pathway that involves ion flux [6]. Initial studies showed that PTX affects JNK activation through a mechanism that involves sodium influx [17]. A later study conducted in rat fibroblasts suggested that PTX stimulates JNK activation through a mechanism that involves potassium efflux [41]. It was also demonstrated that PTX-stimulated signals are transmitted to JNK through the activation of a protein kinase cascade, so that the induction of ion flux by PTX results in the activation of MEK4 (MAPK kinase 4) which phosphorylates and activates JNK [42-45]. Collectively, the JNK MAPKs as an evolutionarily-conserved family appear to be important mediators of PTX-stimulated signals. Noteworthy in this regard is the involvement of JNK3 (MAPK10) in these signaling events and has been verified by our JNK3 protein kinase inhibition experiment showing that the repression of the JNK3 expression is essential for the enhancement of PTX toxicity in cancer cells.

In conclusion, we have demonstrated that head and neck cancer cells and xenografts are more sensitive to PTX than normal cells. Because PTX binds to cell surface receptors present on malignant and benign cells, and acts more effectively upon HNSCC cells, there is a need to pay more attention to this natural product to further define the way of its optimal potential use which may extend our knowledge of the biology of head and neck cancer.

## Competing interests

The authors declare that they have no competing interests.

## Authors’ contributions

T.G. performed the experiments and wrote the manuscript. L.B. purified and isolated the palytoxin and analyzed its toxic effect. E.S.Q. performed the experiments with the control mice and discussed results. P.A. supported the experiments and conducted data analysis. M.H. gave constructive support, collected the data and wrote the paper. All authors discussed the results at all stages. All authors read and approved the final manuscript.
